# Supplementary material for: Reduced monocyte proportions and responsiveness in convalescent COVID-19 patients
Source: Front Immunol. 2024 Jan 4;14:1329026. doi: 10.3389/fimmu.2023.1329026 (PMC10797708; doi:10.3389/fimmu.2023.1329026)
Supplement: Supplementary file 1 [file DataSheet_1.docx]

Supplementary Material

Reduced Monocyte Proportions and Responsiveness in Convalescent COVID-19 Patients

Eugene V. Ravkov, Elizabeth S.C.P. Williams, Mark Elgort, Adam P. Barker, Vicente Planelles, Adam M. Spivak, Julio C. Delgado, Leo Lin, Timothy M. Hanley^*^

*** Correspondence:** Corresponding Author: timothy.hanley@hsc.utah.edu

# Supplementary Figures and Tables


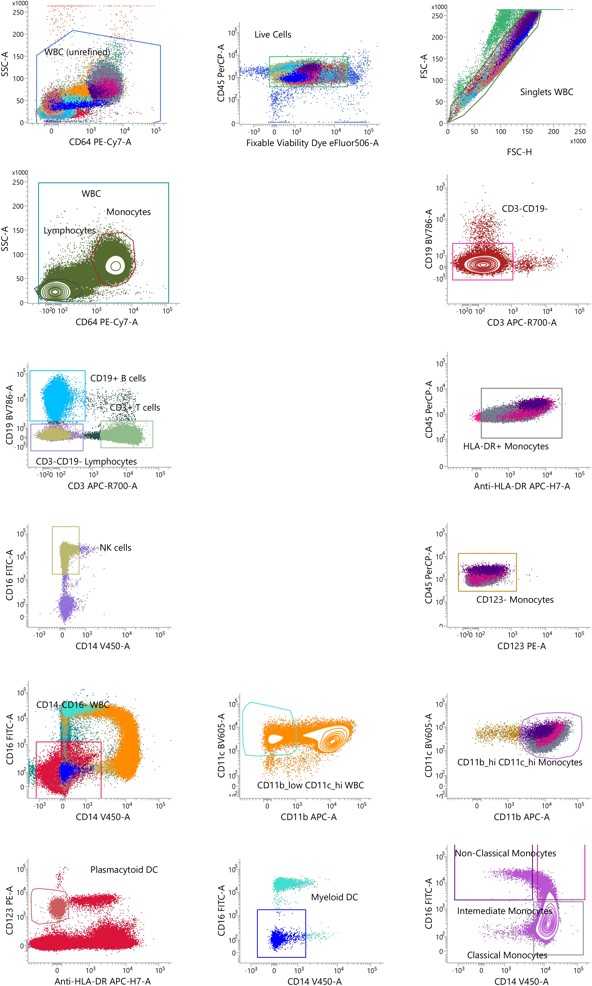

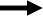

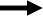

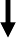

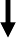

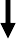

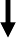

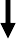

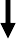

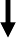

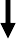

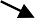

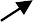

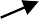


Supplementary Figure 1. Flow cytometry gating strategy for identification of monocytes, lymphocytes, myeloid dendritic cells (mDCs), and plasmacytoid dendritic cells (pDCs).


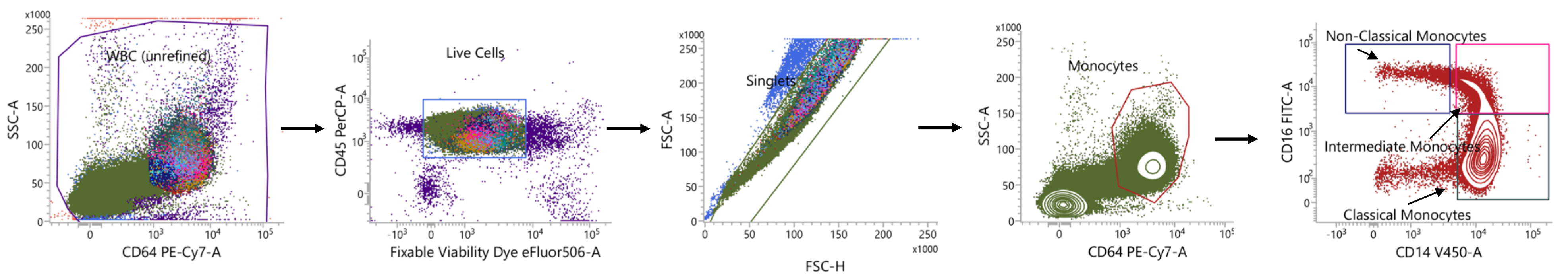


Supplementary Figure 2. Flow cytometry gating strategy for evaluation of monocyte activation markers in monocyte subsets.


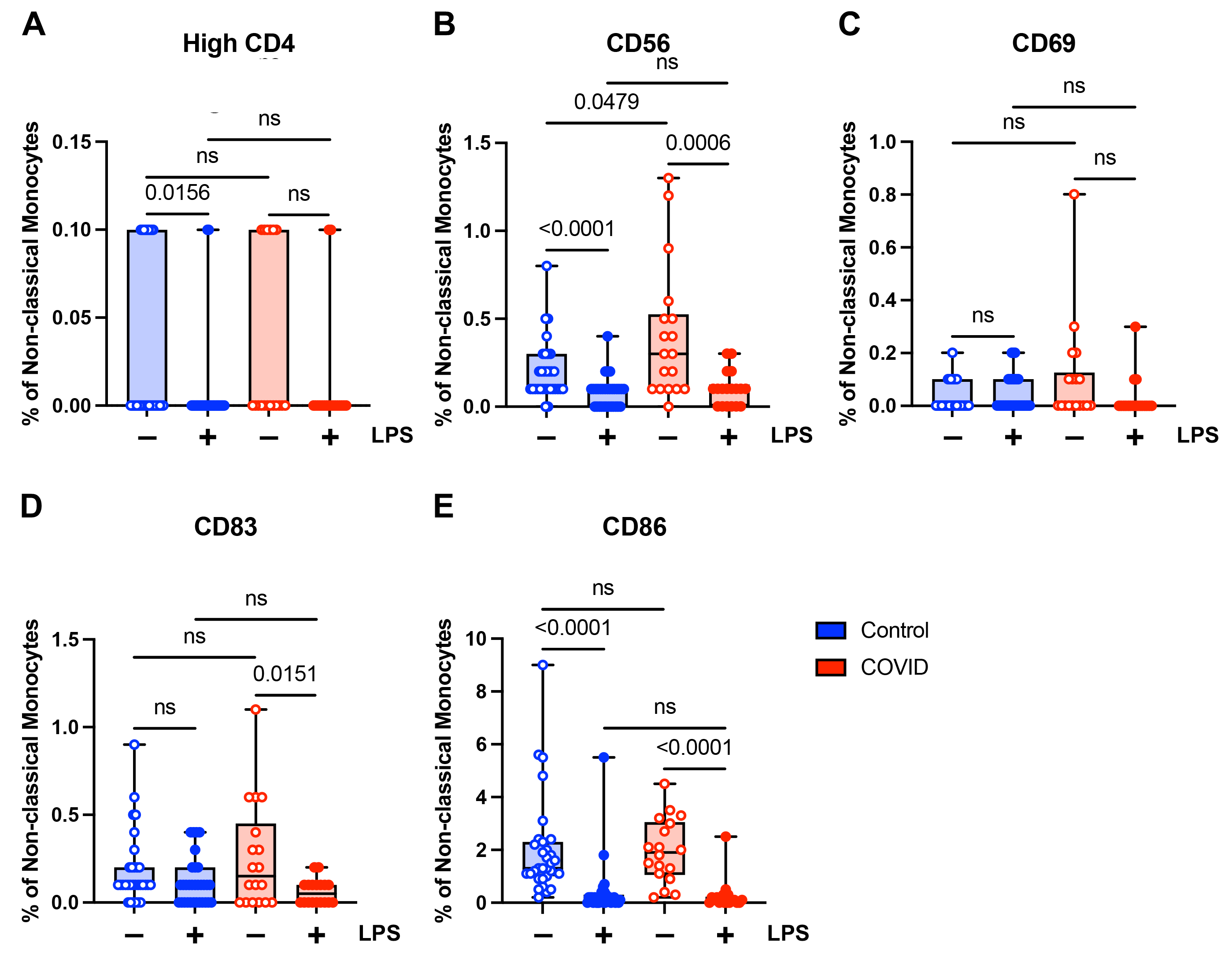


Supplementary Figure 3. Activation marker expression in non-classical (CD14^lo^ CD16+) monocytes. (A) CD4 percentage, (B) CD56 percentage, (C) CD69 percentage, (D) CD83 percentage, and (E) CD86 percentage (of non-classical monocytes) in convalescent COVID-19 (red, n=18) and control (blue, n=31) groups in the absence (empty) or presence (filled) of 100 ng/mL LPS. Comparison between groups (control vs. COVID-19) Mann-Whitney U test. Comparison within groups (untreated vs. LPS-treated) Wilcoxon ranked test. ns, not significant.


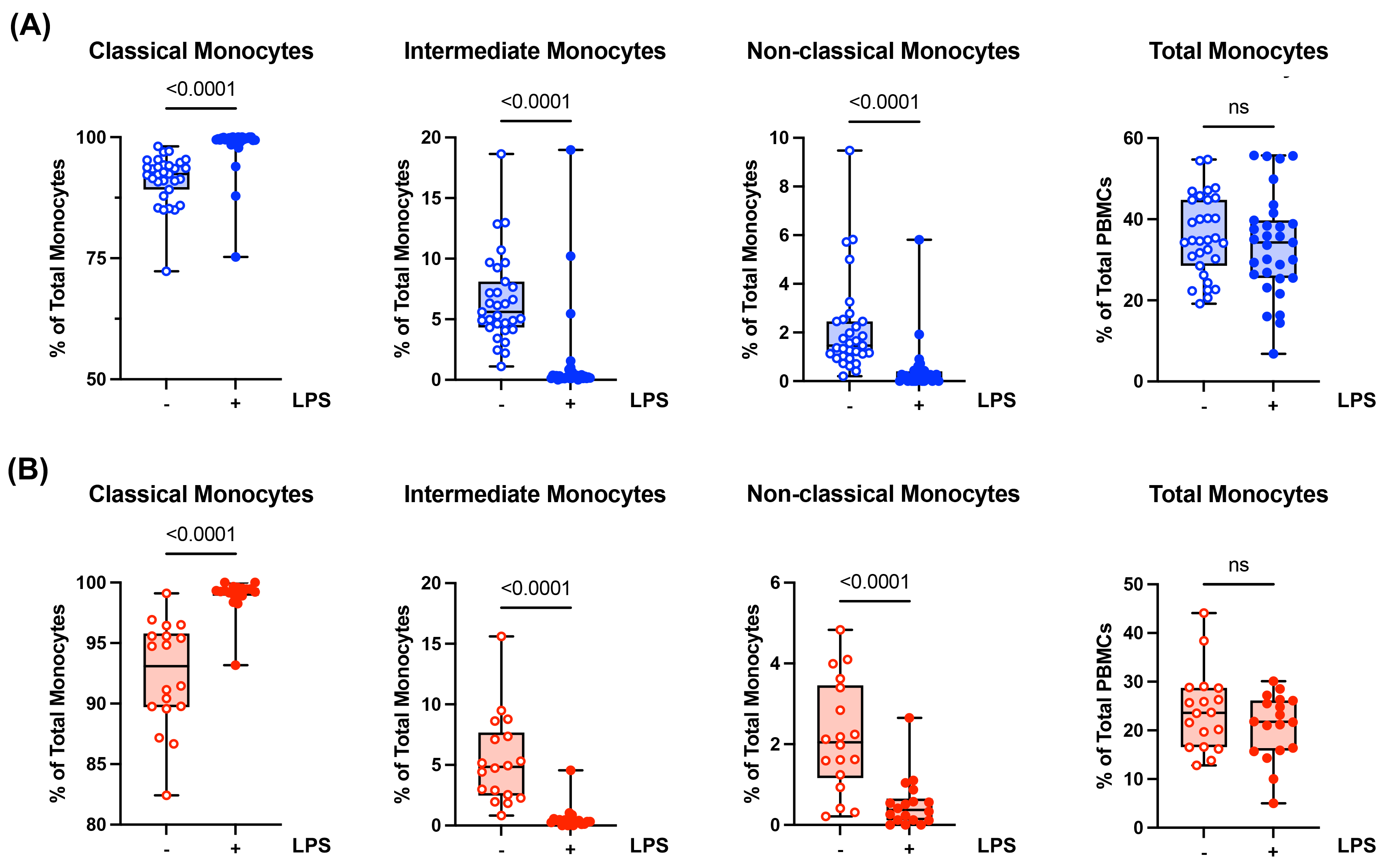


Supplementary Figure 4. Monocyte subset proportions change in response to LPS stimulation. (A) Percentages of classical, intermediate, non-classical, and total macrophages from uninfected control subjects (n=31) in the absence (empty) or presence (filled) of 100 ng/mL LPS. (B) Percentages of classical, intermediate, non-classical, and total macrophages from convalescent COVID-19 patients (n=18) in the absence (empty) or presence (filled) of 100 ng/mL LPS. Comparison within groups (untreated vs. LPS-treated) Wilcoxon ranked test. ns, not significant.
